# Nutrition and Green Nephrology—not only for health but also for compensation: the water footprint and its hamburger equivalent

**DOI:** 10.1093/ckj/sfag320

**Published:** 2026-09-14

**Authors:** Giorgina Barbara Piccoli, Julia Nava, Barbara Romano-Andrioni, Marta Arias-Guillén, Federica De Martino, Junelle Speller, Massimo Torreggiani, Claudia D’Alessandro, Carla Maria Avesani, the European Renal Nutrition Working Group of the European Renal Association, Giorgina Piccoli, Julia Nava, Bárbara Romano, Marta Arias-Guillén, Federica Demartino, Junelle Speller, Massimo Torreggiani, Claudia D Alessandro, Carla Avesani

**Affiliations:** Néphrologie et dialyse, Centre Hospitalier Le Mans, Le Mans, France; Instituto Mexicano del Securo Social, Mexico City, Mexico; Endocrinology and Nutrition Department, Hospital Clinic Barcelona, Barcelona, Spain; Renal Transplantation and Nephrology Department, Hospital Clinic Barcelona, Barcelona, Spain; Freelance, expert patient, Turin, Italy; Expert patient, Chicago, IL, USA; Néphrologie et dialyse, Centre Hospitalier Le Mans, Le Mans, France; Department of Clinical and Experimental Medicine, University of Pisa, Nephrology, Dialysis and Transplantation unit, Pisa, Italy; Division of Renal Medicine, Baxter Novum, Department of Clinical Science Intervention and Technology, Karolinska Institutet, Stockholm, Sweden

**Keywords:** carbon footprint, diet, low protein diet, plant-based food, sustainable nephrology

## Abstract

Nutrition is recognized as a cornerstone of chronic kidney disease (CKD) prevention and management, while healthcare sustainability has become a major priority in nephrology. These two challenges converge because dietary choices influence both kidney health and the environmental footprint of care. In this narrative review, enriched by the perspectives of patients who experienced CKD, dialysis, and kidney transplantation, we discuss how nutritional interventions can simultaneously improve clinical outcomes and reduce the carbon and water footprint associated with kidney care. We summarize current evidence supporting healthy plant-dominant dietary patterns across the CKD continuum, from disease prevention to conservative management, dialysis and transplantation. We highlight the emerging shift from nutrient restriction toward food quality, emphasizing minimally processed plant-based foods, moderation of animal protein intake and individualized nutritional counseling. The review also discusses the evolving evidence challenging traditional dietary paradigms in advanced CKD and dialysis, particularly regarding protein, potassium and phosphate management. To illustrate the environmental implications of nutritional choices, we introduce the concept of the “hamburger equivalent,” comparing the annual water footprint of hemodiafiltration with that required for beef production. Rather than promoting guilt or restricting the choice of effective kidney replacement therapies, this concept illustrates how healthier dietary habits may help offset part of the unavoidable environmental burden of kidney care. Throughout the CKD journey, personalized nutritional counseling that respects patients’ cultural preferences and values may improve adherence, quality of life and clinical outcomes while contributing to planetary health. Integrating sustainable nutrition into routine nephrology practice therefore represents an opportunity to align high-quality patient care with environmental stewardship.

## Graphical Abstract

**Graphical Abstract:**
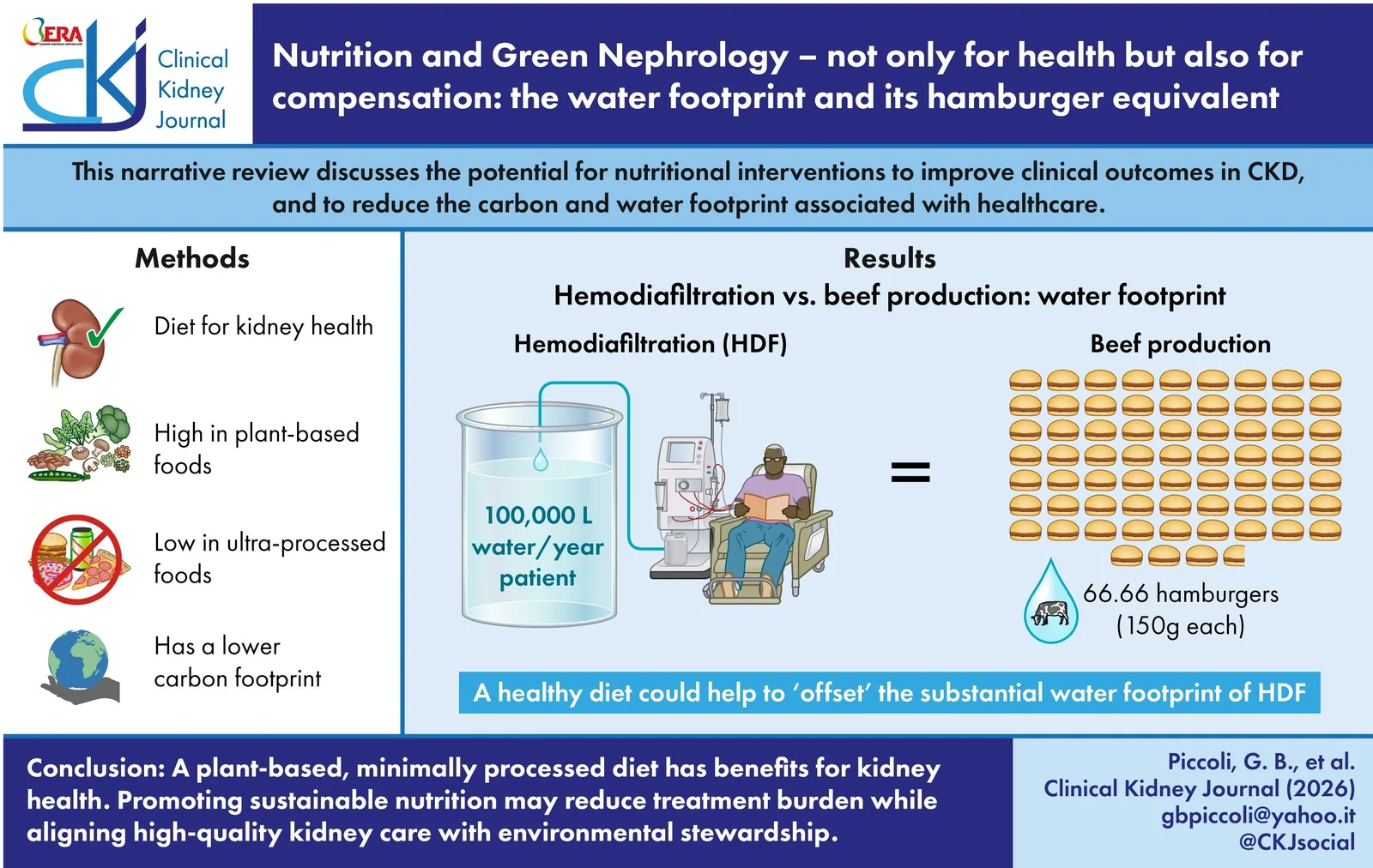

## INTRODUCTION

The healthcare system accounts for up to 5% of the carbon footprint in high-income countries (HICs), while being substantially lower in low- and middle-income countries (LMICs) due to the paucity of infrastructures and funding [1, 2].

Conversely, it has been estimated that about 30% of the total carbon footprint is attributable to the food industry. Nutrition and lifestyle are key contributors to the development of many non-communicable diseases, including those that represent risk factors for chronic kidney disease (CKD). Consequently, whenever CKD is considered from the perspective of cost, sustainability or ethics, nutritional issues must also be addressed.

Population aging, observed in both HICs and LMICs, is mainly linked to better care of acute diseases in LMICs, while in HICs it is largely due to the longer survival of individuals living with chronic conditions, including CKD (prevalence estimated at 10%–14% in highly resourced settings), diabetes (with a similar prevalence, depending on the definitions used), hypertension (affecting up to 30% of the population and increasing with age) and cardiovascular diseases (affecting more than 10%–15% of the population, once again according to the definitions chosen) [3–6]. Obesity is not uniformly cited in this context, but it is increasingly recognized as a non-communicable disease whose prevalence is steadily rising worldwide [7].

Because several of these conditions may coexist, it has been estimated that about 40% of the overall population—especially in countries with long life expectancy—is affected by one or more non-communicable diseases requiring chronic care.

These conditions are closely interrelated, and nutrition represents both a contributing factor and a shared therapeutic tool. Accordingly, strategies for prevention and nutritional management of diabetes, obesity, cardiovascular disease and CKD follow common pathways.

The ecological benefits of promoting healthy dietary patterns are substantial. Such diets are generally environmentally sustainable, as they are predominantly plant-based, rely on fresh and minimally processed foods (often requiring less transportation), include healthy sources of fats, and limit ultra-processed and packaged products. The value of “planetary diets” is increasingly acknowledged not only by scientists, but also by lay societies and media [8, 9]. Even trying to avoid the oversimplification of a “carbon tunnel vision,” and considering also water footprint and waste management, reduced consumption of red and processed meats contributes to a lower carbon and water footprint, while minimizing packaged food intake reduces plastic waste and decreases exposure to plastic-derived contaminants, which are increasingly recognized as health threats, including to kidney health [10–12].

In this review, enriched by the narrative of two patients with different experiences, we will discuss how nutritional management not only contributes to the first and absolute goal of improving patient’s health, but may also represent a way to attain a neutral balance in kidney care.

## A PATIENT’S POINT OF VIEW

### Federica’s experience

I have been living with a kidney transplant for more than 20 years. As a woman from Southern Italy, I have always appreciated Mediterranean cuisine, and from my short pre-dialysis phase I learned that a diet too rich in proteins could be detrimental to kidney function. My transplant was a gift, and I have done everything possible to preserve it. From the beginning, perhaps also because my donor was much older than me, my serum creatinine stabilized at around 2 mg/dL.

Attitudes toward what is now referred to as a plant-based diet have evolved over time. When I was a newly transplanted patient, I was strongly advised to avoid sugars, while meat consumption was encouraged to counterbalance the effects of steroids and immunosuppressive therapy.

Since a young age, including during dialysis, I have enjoyed going to the gym and weight training. This habit has likely contributed to maintaining good muscle mass, even though I have gained some weight over time. I have rarely discussed my dietary habits with the physicians at my transplant center, and I was never referred for formal dietary counseling. However, based on my previous experience with an earlier physician, I adopted a diet providing 0.6–0.8 g of protein per kilogram per day. Only recently, one of my current physicians acknowledged that this dietary approach may be one of the reasons for my long-term preservation of kidney function. As a patient with longstanding experience of kidney disease, I would welcome greater patient empowerment and respect for individual choices. Diet has never felt like a constraint to me; rather, it has been a valuable tool, and as such I believe it should be offered to all patients. Foods that are natural and minimally processed—those that come directly from the earth—seem to me both a healthy and sustainable choice. Having lived with illness from a young age, my primary concern has always been my health rather than the health of the planet. However, I am deeply aware of life’s fragility, and knowing that my choices may also be environmentally sustainable makes me even more proud and fulfilled.

### The hamburger-equivalent issue: a healthy diet for carbon footprint compensation

Nowadays, when checking in for an airplane flight, passengers are invited to offset the carbon footprint of their journey by supporting environmentally friendly initiatives. This practice is based on the recognition that certain activities, such as air travel, have an intrinsic carbon footprint that cannot be fully eliminated but may be partially compensated through positive, sustainability-oriented actions.

In nephrology, nutrition remains a “Cinderella” component of care, often underemphasized despite its potential impact both on patient’s and planetary health [10–12]. A deeper integration of nutritional strategies into clinical practice could contribute to offsetting the carbon and water footprint associated with kidney care. In the green dialysis context, while some experts have proposed incorporating sustainability considerations into dialysis modality selection, others suggest that a broader, holistic integration of lifestyle changes is warranted [13]. This perspective supports proactive, patient-centered choices and encourages engagement in lifestyle and dietary changes without fostering guilt or stigmatization, or impacting on the choice of the preferred dialysis modality.

The main characteristics of a patient- and planet-friendly diet from prevention of CKD to kidney replacement therapy will be discussed in the following paragraphs.

Here, to highlight the potential of “compensation” through a healthy diet, we would like to refer to the example of the water footprint of hemodiafiltration (HDF) (Fig. 1). Water consumption is classically estimated as about 350–800 L per session in hemodialysis and it is estimated that HDF increases the need by further 50 L per session (25 L of reinfusate, produced through a reverse osmosis with a 50% yield). Hence, some authors are concerned about the ecologic costs of expanding this dialysis modality, even though better survival has been reported, at least in selected populations [14].

Notwithstanding the growing interest for reducing the water footprint through modulation of dialysate flows, which according to a recent *in vitro* and *in vivo* study may offset the differences between water footprint of hemodialysis and HDF without affecting the dialysis efficiency [14–16], we compared the annual water footprint of HDF using a “worst-case scenario” of 500 L of water consumption per session (details in Fig. 1) and compared it with that of red meat consumption [17, 18]. Our calculations, while approximate, suggest an equivalence to the water required to produce approximately 66.66 beef hamburgers (150 g each) (Fig. 1). These estimates are inherently variable, since for instance, water consumption differs substantially between grazing and industrially farmed livestock [19, 20]. Nevertheless, the broader concept remains robust: adopting a healthy, environmentally sustainable diet may help compensate the high water requirements of even the most demanding kidney replacement therapies, and is expected to concomitantly reduce the individual carbon footprint. Such an approach offers a dual benefit: promoting patient health through the choice of the “best” treatments while reducing individual environmental impact, through a healthier diet.

**Figure 1:**
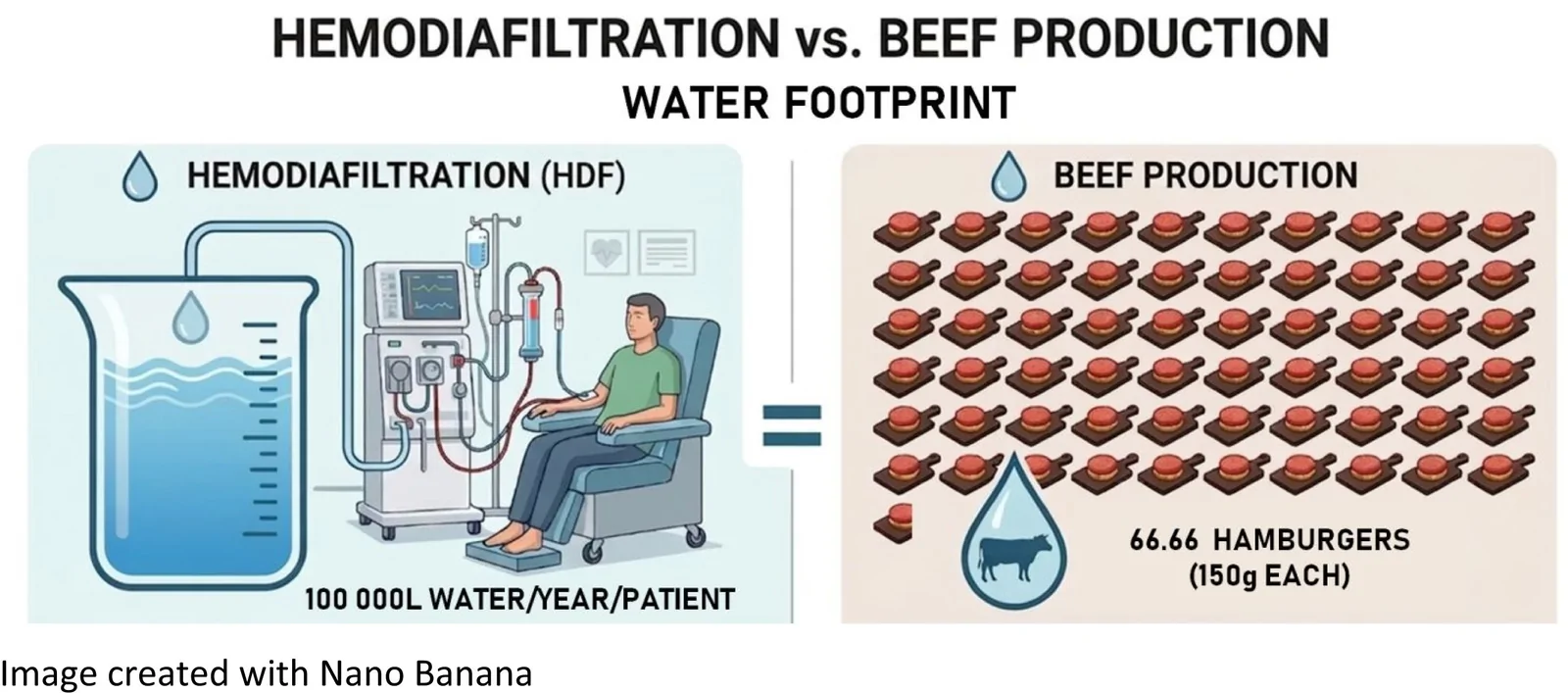
Considering that 1 year of HDF consumes on average 100 000 L of water per patient, and that producing a single industrially made hamburger requires about 1500 L of water, the annual water usage for one patient undergoing HDF is equivalent to the water needed to produce approximately 66.66 hamburgers. Image created with Nano Banana.

Following a plant-based diet, or even a vegan one, for health-related reasons, should not be synonymous with completely banning some food (notably red meat) during all the lifespan, but limiting its consumption to selected occasions. For instance, many management strategies include one or more unrestricted meals per week, to combine healthy approaches with flexibility, and improving patient adherence.

Conversely a plant-based diet is not necessarily a healthy, or a planet-friendly diet. Ultra-processed food is progressively invading the “green” supermarkets shelves, and the carbon footprint of transportation of plant-based food may likewise be very high, and not only in the Northern countries. For example, in France it has been reported that up to 60% of marketed fruits come from abroad [21], and the figure reaches 86% in the United Kingdom [22]. Hence, while it is relatively easy to calculate the water or carbon footprint of some locally produced food, as previously exemplified in the case of red meat, understanding the “net advantage” of a plant-based diet can be very difficult, as the available figures usually consider production, and not for instance overseas transportation.

## NUTRITION, THE PLANET AND PREVENTION OF CKD

Health begins *in utero*, and nutritional derangements or unhealthy nutritional patterns during pregnancy can have lasting effects on both pregnancy outcomes and long-term adult health [23].

Obesity and diabetes are associated with a higher risk of developing hypertensive disorders of pregnancy; poor diabetes control is likewise associated with an increased risk of congenital malformations. Hypertensive women are at risk for superimposed preeclampsia and all these conditions are associated with preterm delivery and being small for gestational age [24, 25], which are associated with reduced nephron endowment and an increased risk of developing hypertension, cardiovascular diseases, obesity and CKD later in life [26]. Furthermore, the presence of maternal CKD is associated with a higher risk of developing adverse pregnancy outcomes, which, in turn, are associated with CKD development [27].

Conversely, population-level data indicate that healthy dietary patterns are associated with improved pregnancy outcomes [28].

Families of children born preterm or small for gestational age should be informed about the increased risk of obesity and hypertension [29]. Although restrictive diets are not recommended, these children may be more susceptible to adverse effects when exposed to energy-dense, high-salt and low-quality foods. Simply acknowledging these risks may, in itself, represent an effective preventive strategy.

From infancy through childhood, diet plays a crucial role in shaping taste preferences. Healthy eating habits are established early in life and are associated with better cognitive development and a lower lifetime risk of obesity [30]. Prevention of CKD largely overlaps with the prevention of cardiovascular diseases, obesity and diabetes. These conditions are closely interrelated, and nutrition represents both a contributing factor and a potential therapeutic tool for all of them. Accordingly, nutritional strategies for CKD prevention, as well as the prevention and nutritional management of diabetes, obesity and cardiovascular disease share common pathways [30].

Since patients with obesity, diabetes or cardiovascular disease are rarely seen by nephrologists before the onset of CKD, greater involvement of the nephrology community in defining and promoting shared preventive strategies is warranted.

## FIRST THINGS FIRST—DEFINING LOW-PROTEIN DIETS AND PLANT-BASED DIETS

Patients with CKD stages 3–5 who are not on dialysis, are not malnourished and meet the energy requirements, are generally advised to follow a diet with the recommended (0.8 g/kg/day of protein) or a lower low-protein content [31]. A low-protein diet is therefore defined as one that contains less protein than the amount recommended for the general population (i.e. aiming to meet the requirements of 97.5% of healthy individuals), which is currently 0.8 g/kg/day [32]. From the energy perspective, a low-protein diet contains a smaller proportion of calories from protein and a greater proportion from fat and carbohydrates. In addition to the quantity of proteins, particular attention is paid to the quality, therefore, in food-based terms, this usually translates into a dietary pattern with more plant-based foods and fewer animal-derived foods. Such a pattern aligns with the concept of a plant-based diet, in which at least 50%–60% of total energy intake comes from foods derived from plants. Examples of plant-based dietary patterns include, among others, the Dietary Approaches to Stop Hypertension (DASH) diet, the Mediterranean diet and the Okinawan diet [33–36]. For patients with CKD, the Plant Dominant (PLADO) and New Nordic Renal Diet (NNRD) approaches have adapted Mediterranean-style principles to the food habits of specific geographic regions, namely the United States and the Nordic countries, respectively [37–39].

Although plant-based diets are often perceived as uniformly healthy, it is important to identify unhealthy plant-based foods which include for example ultra-processed vegan products such as potato chips and similar snack products, sugar-sweetened beverages, fats derived from coconut and hydrogenated oils [40]. While refined grains are not necessarily unhealthy, the advantages of increasing dietary fibers are well established [41]. While theoretically the shorter food-processing chain should increase sustainability, caveats exist on pesticide content, typically concentrated in the seed coat: some pesticides—especially systemic fungicides and some insecticides—can deposit in the wheat seed husk. The bran is the most exposed part and tends to retain more residues than the endosperm, which is why whole-grain flours exhibit more frequent residues, although they remain within safety limits [42].

### Nutrition, CKD care and the low-protein conundrum: what is good for the patient is good for the planet

Once CKD is established, the general principles of healthy eating become even more relevant. Prevention and management of obesity and diabetes acquire additional importance, as they help reduce glomerular hyperfiltration, the main non-immunologic driver of kidney function decline, thereby interrupting a vicious cycle of maladaptive responses [43]. Similarly, the reduced capacity of the diseased kidney to excrete sodium, potassium and phosphate—particularly inorganic phosphate—enhances their toxic potential. This is also true for food additives and preservatives, further reinforcing the dual benefit of limiting ultra-processed foods for both patient health and environmental sustainability. Furthermore, plant–based diets may contribute to a lower phosphate and potassium burden, as phosphorus exists mainly in the form of phytate showing substantially lower intestinal bioavailability, and vegetarian patterns have been associated with lower serum phosphorus and fibroblast growth factor-23 levels in CKD [44].

A particularly debated and kidney-specific issue concerns protein intake. In the context of a cultural and societal shift toward protein- and meat-rich diets, low-protein regimens are often perceived as unpalatable [45]. Although there is no evidence that moderately protein-restricted diets induce malnutrition, when correctly performed and controlled, concerns have been raised about their potential impact on the already fragile quality of life of patients with CKD [46].

Despite these concerns—largely theoretical and not consistently demonstrated—the potential benefits of low-protein diets are considerable. These diets, typically characterized by reduced intake of animal-based foods, are associated not only with stabilization of the kidney function but also with a reduced pharmacological burden, due to the lower phosphate bioavailability and a higher alkaline potential [47]. Notably, the physiological effects of protein restriction—such as reduction in glomerular hyperfiltration, intraglomerular pressure and proteinuria—are similar, and probably additive, to those achieved with established and emerging nephroprotective therapies, including angiotensin-converting enzyme inhibitors, angiotensin receptor blockers and sodium-glucose transporter 2 inhibitors [48].

Their impact on the planet is expected to be lower, as plant-based as well as moderately restricted low-protein diets have a much lower carbon and water footprint than high-protein diets (Fig. 2).

**Figure 2:**
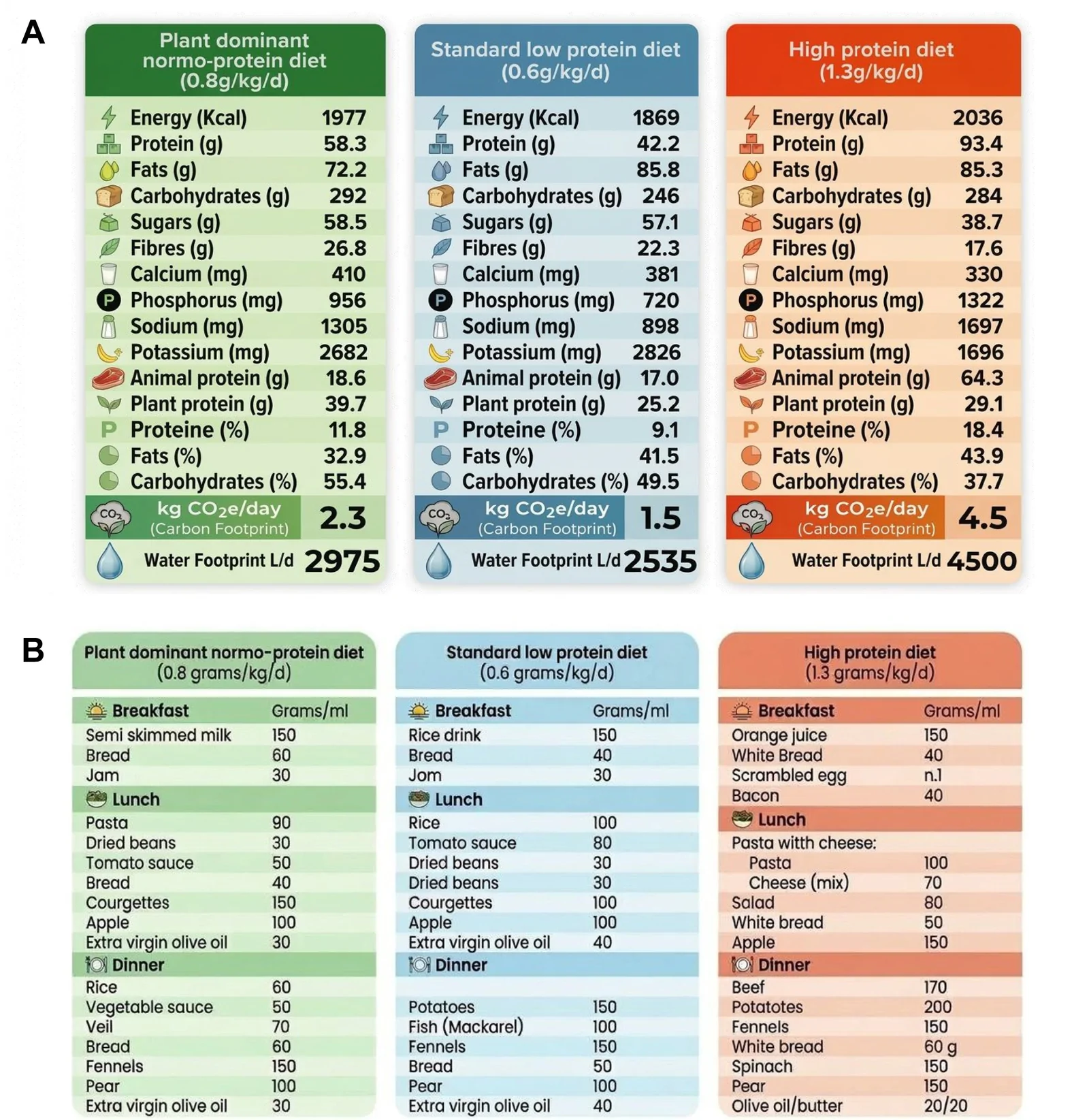
(**A**) Nutritional facts, carbon and water footprint of a plant-dominant diet with a normal content of protein according to World Health Organization recommendations, a standard low-protein diet and a diet with a high protein content with a prevalence of animal protein. (**B**) Composition of a typical day for each diet. Calculations performed for a person weighing 70 kg.

Whether a well-balanced, protein-restricted diet could serve as a surrogate for, or merely a complement to, pharmacological therapy remains an open question. However, indirect support for its efficacy comes from the nutritional management of pregnancy in women with CKD [49]. In this context, where standard nephroprotective drugs are contraindicated, a carefully controlled moderately protein restricted, healthy plant-based diet has been shown to support fetal growth while stabilizing maternal kidney function—once again aligning benefits for patient health and environmental sustainability.

Similar considerations may apply to kidney transplantation, where concerns on muscle mass loss and weight gain, strictly linked to prolonged steroid treatment, have classically led to high-protein prescription, the trends, in the rather neglected field of nutritional management after kidney transplantation, are progressively aligning with those of CKD, with an increase in healthy plant-based approaches, and the avoidance of high protein content [50].

A further issue regards the so-called very low protein diets, which have a protein content of about 0.3–0.4 g/kg/day. While these diets have allowed the best results in terms of dialysis-free survival, and their advantages are acknowledged by both the KDIGO and KDOQI guidelines, they are not easy to follow and the two most important trials on this regard, Garneata’s [51] and Brunori’s in elderly patients [52], were able to enroll only between 15% and 30% of the potential candidates. These diets are typically vegan, and hence easily designed in a planet-friendly way; however, as a note of caution, they require supplementation with a mixture of amino acids and ketoacids, whose carbon footprint is not yet established.

### Challenging the diet dogmas in patients with advanced CKD and on dialysis: we need to rethink protein, phosphate and potassium content

While a detailed discussion of dietary management in patients with kidney failure is beyond the scope of this review, the traditional paradigm of a diet rich in animal proteins and highly restricted in plant-based foods is increasingly being challenged, and a diet both richer in plant-based food and with a limited content of animal proteins is increasingly being promoted in patients living on dialysis, and, as for potassium and phosphate, also in patients living with severe kidney function impairment [44, 47, 53].

Several factors may contribute to the shift towards a moderate protein intake in dialysis, including improvements in dialysis efficiency and in the management of associated conditions—such as anemia, metabolic acidosis, and mineral and bone disorders—which together mitigate the hypercatabolic effects of the dialysis sessions.

A diet rich in animal derived food, and particularly in red meat, promotes acidosis and hyperphosphatemia, and may expose patients with limited or no residual kidney function to the negative effects of the additives present in ultra-processed meats [44, 47, 53–55].

The lower availability of phosphate contained in plant-based food may allow more dietary freedom, while, once more, attention shifts towards the avoidance of ultra-processed food, not only based on their often high potassium or phosphorus content, but also on its rapid and almost complete bioavailability [44].

As for potassium, recent literature challenges the traditional paradigm of potassium restriction in patients living with CKD and on dialysis. Emerging data suggest that potassium derived from whole-plant foods has a lower bioavailability and does not translate into higher serum potassium levels, or menacing hyperkalemia episodes, in particular when consumed within a high-fiber dietary pattern [56, 57]. The inclusion of fresh vegetables and of omega-3 polyunsaturated fatty acids is supported by recent randomized evidence of cardiovascular benefit, specifically in hemodialysis patients [58].

In addition, the adoption of incremental dialysis strategies and the attention to the preservation of residual kidney function have further supported a re-evaluation of dietary recommendations, with different policies, ranging from systematically combining low-protein diets and once-twice weekly dialysis (the “Pisa” system) to offering the flexible option of a moderately protein-restricted diet in patients on once or twice weekly dialysis (the “Le Mans” system) [59, 60].

While only long-term data may allow conclusions on the safety and advantages of these policies, no data as far suggests a nutritional disadvantage linked with moderating protein intake in dialysis patients with good nutritional status, particularly on incremental dialysis.

## PATIENT PREFERENCES AND “DIETS” DURING THE CKD CONTINUUM

The etymological meaning of diet, from the Greek dieita and Latin diaeta is “way of life,” conveying a comprehensive concept of lifestyle, has partially been lost in the last century.

In its broader meaning, the term diet embraces patients’ life and preferences, and not merely some (often restrictive) prescriptions. Re-discovering this concept is important when a “diet” is prescribed in an individual with a chronic disease, as it acknowledges both the need for a personalized approach and the importance of flexibility, allowing personalized modulation, occasional exceptions, adapting diets to needs and goals, and periodically reconsidering goals, needs and preferences. These issues are relatively unexplored in nephrology [61].

Unrestricted meals, which some of the authors of this review allow up to one to three times per week, represent a strategy not only for avoiding tensions between restrictive diets and quality of life, but also potentially effective in supporting an educational approach to nutrition [62].

Over the course of CKD, while some issues remain the same, like adequacy of energy intake, and food quality, other’s may change according to the residual kidney function and the drug therapy. This continuous clinical modulation should be associated with continuous discussion with the patients, ensuring understanding and sharing prescriptions, if possible, in the form of “educational hints” more than “fixed rules.”

In such an educational pathway, highlighting the “secondary” advantage for the planet both of retarding dialysis and of wisely choosing plant-based food may help reinforcing adherence, and engaging into discussion.

## A PATIENT’S POINT OF VIEW

### Junelle’s perspective

For nearly two decades living with kidney disease, I have experienced nutrition from multiple perspectives: as a patient with CKD, as someone who spent years on dialysis, and now as a transplant recipient for the second time. Growing up in a Jamaican household in Chicago, food was never just sustenance—it was culture, family and identity. Mangoes, ackee, plantain, yam and gungo peas were among the many foods that were foundational to my childhood and remain important to me. Learning to navigate their restriction while on the “dialysis renal diet” I was prescribed was one of the most difficult aspects of managing my disease.

Before dialysis, I ate primarily plant-based foods, a choice rooted in both cultural tradition and a genuine concern for sustainability. When I began peritoneal dialysis, my care team encouraged me to increase my protein intake. Much of that added protein came from meat, because beans—long a staple of my diet—were often discouraged because of concerns about phosphorus intake. The shift felt like a double loss: I was moving away from the foods that connected me to my heritage and an approach to eating that felt right for the planet. That experience taught me that nutritional recommendations, however well-intentioned, are not neutral. They land inside a life, a culture and a set of values.

What I have come to understand is that the most effective dietary guidance is not built solely on restriction. The nutritional needs of a person with kidney disease change over time, and recommendations appropriate at one stage may no longer fit at another.

For many patients, the challenge is not only understanding what is healthy but also finding ways to integrate those recommendations into work, family life, cultural heritage, personal values and the everyday realities of living with a chronic condition.

The most successful conversations I have had with clinicians—primarily dietitians and nurses, who were most often present when diet was discussed—were those that treated nutrition as a partnership rather than a prescription. When patients understand the purpose behind a recommendation and are invited to participate in the decision-making process, adherence becomes easier, and quality of life improves.

Today, four years into my second transplant, my dietary restrictions have lessened considerably. Grapefruit and pomegranate are the main exceptions to avoid their interactions with my immunosuppressive medications. I have found my way back toward a more plant-centered way of eating, and with it, a renewed sense that my choices can serve both my health and broader environmental sustainability. That simplification feels like a kind of freedom and a reminder of how much the landscape of living with kidney disease can shift.

From my perspective, a collaborative approach to nutrition is equally important whether the goal is to preserve kidney function, support life on dialysis, protect a transplant or reduce our collective impact on the planet. Lasting change occurs when dietary choices support both health and daily life and leave room for the foods that connect us to who we are.

## WORKING CONCLUSIONS

In renal nutrition, what benefits the patient—namely a plant-based diet rich in fresh foods and low in ultra-processed products—is also beneficial for the planet. Meanwhile, some have data started supporting that a “planetary diet” is also beneficial for the kidney function. Its promotion is therefore both clinically sound and environmentally sustainable.

Beyond encouraging the broader use of a plant-based healthy diet, to be tailored and incorporated into patients’ daily lives, raising awareness of the advantages of healthy and sustainable food habits may help offset the unavoidable environmental burden associated with the treatment of kidney disease, in particular kidney failure.

Reducing the treatment burden, be it retarding or reducing the need for dialysis, or limiting the number of pills, by means of sustainable dietary practices represents an opportunity to align high-quality patient care with responsible stewardship of environmental resources.

## Data Availability

No new data were generated or analyzed in support of this research.
